# Bibliography

**Published:** 1906-01-15

**Authors:** 


### BIBLIOGRAPHY.

LONG'S DENTAL MATERIA MEDICA AND THERAPEUTICS. A text-book of Dental Materia Medica, Therapeudics and Prescription Writing. For students and practitioners of dentistry. By Eli H. Long, M. D., Professor of Materia Medica and Therapeutics in the Dental and Medical Departments of the University of Buffalo, New York. New (2d) edition enlarged and revised to accord with the new United States Pharmacopœia. In one octavo volume of 298 pages with seven engravings and eighteen colored diagrams. Cloth, $3.00 *net*. Lea Brothers & Co., Publishers, Philadelphia and New York, 1905.

A second edition of this most valuable text-book is good evidence that this work by a medical man has met a need in teaching that has never been as fully accomplished by similar books from dental teachers. It is true that we miss in many places the stress that a technical man would put on the proper selection and manner of using certain remedies which do not appeal to a medical or scientific writer. The chapters on anesthesia, antizymatics, counter irritants, astringents and stimulants are very well done indeed. The several charts showing nerve reflexes etc., are a great help in comprehending the indirect reactions of remedies. The tables of poisons with their antidotes and the list of up-to-date drugs make an invaluable addition to such a work. We are pleased to have this work brought down to date and believe it will be fully appreciated by the profession. The chapter on bleaching agents should be given more extensive treatment, but this is a subject that is so little studied by the average practitioner, that perhaps the elements only will suffice. We most cordially commend the book to students and practitioners, and express to Dr. Long our thanks for his faithful work.

The American Text-book of Operative Dentistry. In contributions by eminent authorities. Edited by Edw. C. Kirk. Chapters written by Edward H. Angle, Henry H. Burchard, Calvin S. Case, Dwight M. Clapp, William Crenshaw, M. H. Cryer, Edwin T. Darby, C. L. Goddard, S. H. Guilford, Joseph B. Head, Louis Jack, Edward C. Kirk, F. B. Noyes, Louis Ottofy, C. N. Pierce, J. D. Thomas, A. H. Thompson, James Truman. New (3d) edition, enlarged and thoroughly revised. In one octavo volume of 899 pages with 875 illustrations. Cloth, $6.00, *net;* leather, $7.00 *net;* half-morocco, $7.50 *net.* Lea Brothers & Co., Publishers.

The new edition of this work which is perhaps the most pretentious book ever published on the subject of "Operative Dentistry," is a sort of cyclopedic treatment of this department of practice. The gentlemen selected for presenting the different topics have each done his work largely from his own view point, consequently the book lacks somewhat of continuity and singleness of statement. Whether this is the best method to adopt in preparing a class text-book or not is a question that should be thoroughly studied. The probabilities are that the text book by a single author will embody a more systematic statement of the principles with the least amount of repetition, and be therefore the more readily assimilated by the student. In this work there are at least four statements of the various forms of cavity preparation, and while there is no apparent contradiction of consequence, the student will never get a firmly fixed idea of any method from reading the different methods from the same book. Of course, this objection can be overcome by the teacher in the clinic and class room, but it makes unnecessary labor to do this, and there is nothing special to be gained by such a confused presentation. We think the whole work could be wonderfully simplified by cutting down the chapter by Dr. Noyes on tooth histology and following this with a carefully illustrated system of cavity preparation by such a man as Dr. G. V. Black. By this we don't wish to discredit the contributions of the writers in the present work on cavity preparation, because their work is well done, in fact it is too well done. The present work is greatly improved by the contribution of Dr. E. H. Angle, on the subject of Orthodontia. We, however, do not see why this subject should be included in a work on operative dentistry, but since it is we think the editor has wisely chosen his authors, as they have contributed as good a statement of modern orthodontia as may be found in any text book. The new chapter on the use of matrices, by Dr. Crenshaw, is timely and well done. The value of the matrix for making proximal cavities with the plastic filling materials is not recognized, as it should be by the average practitioner. It would do every practitioner a lot of good to read this chapter carefully. He would find it practicable to make restorations of lost tooth tissue more easily and definitely than he is now doing. He would also get some valuable pointers on making cavity preparations for the use of the matrix. The subject of porcelain inlays is very well presented, but we are disappointed that the gold inlay is not more fully described and illustrated. The gold inlay, in our judgment, is destined to come strongly into favor in the next few years. There is nothing said of the subject of Dental Prophylaxis, which is certainly a branch of operative dentistry, and one too that is coming swiftly into favor. It is destined, in our judgment, to revolutionize the practice of dentistry, and we regret that Dr. Smith or Dr. Rhein was not asked to contribute a chapter on this important subject. The book is one that we ought be proudof in spite of these lapses, as it is well edited and published in good style.

